# Evaluation of the effectiveness of GASMAN anesthesia simulation software combined with case-based learning versus traditional lecture-based learning in inhalation anesthesia education

**DOI:** 10.3389/fmed.2024.1472404

**Published:** 2025-01-06

**Authors:** Chao Chen, Shengfeng Yang, Xinglong Xiong, Yewei Shi, Zhenyan Zhu, Jing Shi

**Affiliations:** ^1^Department of Anesthesiology, The Affiliated Hospital of Guizhou Medical University, Guiyang, Guizhou, China; ^2^School of Anesthesiology, Guizhou Medical University, Guiyang, Guizhou, China; ^3^Department of Nursing, The Affiliated Hospital of Guizhou Medical University, Guiyang, Guizhou, China

**Keywords:** GASMAN software, inhalation anesthesia, undergraduate students, teaching, case-based learning

## Abstract

**Objective:**

To evaluate the effectiveness of integrating GASMAN anesthesia simulation software with case-based learning (IGC) compared to traditional lecture-based learning (LBL) in teaching inhalation anesthesia to undergraduate anesthesiology students.

**Methods:**

Fourth-year students from two academic years (2022, *n* = 110; 2023, *n* = 131) enrolled in a five-year anesthesiology program were assigned to either traditional lecture-based learning (LBL) or IGC groups. The LBL group received traditional lectures using PowerPoint slides, while the IGC group engaged with GASMAN anesthesia simulation software (a tool designed for anesthesia simulation and gas monitoring) combined with case-based learning. The cases used in the IGC group were structured around realistic clinical scenarios, simulating real-world challenges in inhalation anesthesia. These scenarios were integrated with the GASMAN software to provide interactive simulations, enhancing students’ understanding of pharmacokinetics and pharmacodynamics. Teaching effectiveness was evaluated through expert assessments and student feedback, with learning outcomes compared via post-course assessments.

**Results:**

The IGC group scored significantly higher in student evaluations in areas such as comprehending and mastering theoretical knowledge, resolving clinical challenges, nurturing clinical reasoning, increasing learning interest, enhancing learning efficiency, consolidating memory, improving analytical skills, and refining application proficiency (adjusted *P* < 0.001), however, there were no significant differences between the two groups in the improvement of practical skills. Post-course test scores were also higher in the IGC group for both total post-course test and subjective questions scores (adjusted *P* < 0.001), though no difference was found for objective question scores. After applying false discovery rate (FDR) correction, expert evaluation scores showed no significant differences between the two groups.

**Conclusion:**

The integration of GASMAN software with case-based learning significantly enhances the quality of inhalation anesthesia education by improving student engagement, critical thinking, and conceptual understanding. This approach demonstrates promise for advancing clinical education, although further research is needed to evaluate its long-term impact.

## 1 Introduction

Inhalation anesthesia is a fundamental technique in clinical anesthesia, involving the administration of volatile anesthetics via the respiratory tract, which then diffuse into the bloodstream through the alveoli and reach the brain to induce general anesthesia. This method is among the most commonly utilized in clinical settings (1). Mastering the complexities of inhalation anesthesia is essential for anesthesiology students, as the subject involves intricate pharmacokinetic and pharmacodynamic concepts. These include understanding the absorption, distribution, metabolism, and excretion of volatile anesthetics, which are critical to ensuring safe and effective patient care (2). Such complexities, particularly in pharmacological principles, are often difficult for beginners to grasp, leading to common challenges in understanding how inhalation anesthetics interact within the body (3, 4). This gap in comprehension can result in students lacking confidence during clinical practice and internships.

Traditional lecture-based learning (LBL), which remains a common instructional method in medical education, often struggles to actively engage students or to adequately develop their clinical reasoning skills. These limitations are particularly pronounced in complex subjects like inhalation anesthesia, where passive information delivery may not effectively convey the intricacies of pharmacokinetics and pharmacodynamics. Consequently, educators may find themselves re-explaining concepts multiple times to facilitate deeper understanding (5, 6).

To address these challenges, case-based learning (CBL) has been introduced as a student-centered pedagogical strategy that employs real-world clinical scenarios. In CBL, typical or challenging cases are selected to align with specific learning objectives. These cases serve as a catalyst for guided discussions, encouraging students to engage actively, apply theoretical knowledge, and develop problem-solving skills in a context that mirrors clinical practice (7). In the field of anesthesiology, CBL is particularly beneficial because it allows students to encounter and work through the complexities of anesthesia management in a controlled environment, thereby enhancing engagement and fostering clinical reasoning (8). Research has shown that CBL’s emphasis on active participation and contextual learning leads to improved knowledge retention, critical thinking, and readiness for real-world clinical scenarios (9).

To further enrich CBL in anesthesiology education, the GasMan anesthesia simulation software offers an interactive and dynamic platform that simulates the pharmacokinetics and pharmacodynamics of volatile anesthetics (10). Developed by Professor Philip, GasMan models the trajectory of anesthetics as they transition from the vaporizer to the respiratory system, alveoli, bloodstream, target organs, and back for exhalation (11, 12). This detailed simulation assists students in visualizing and understanding the complex dynamics of inhalation anesthesia, which are often abstract and challenging to conceptualize in traditional learning settings (13). Additionally, GasMan allows for the replication of diverse pathological and physiological conditions—such as severe obesity, heart failure, and restrictive ventilatory dysfunction—that are difficult to simulate in clinical practice. These scenarios provide invaluable learning opportunities that would otherwise be inaccessible in a conventional classroom (14–17). The integration of GasMan with CBL in the instruction of inhalation anesthesia enhances the educational experience by enabling students to engage with complex cases in a simulated environment. The software’s interactive simulations help bridge the gap between theoretical concepts and practical application, allowing students to analyze patient data, make informed clinical decisions, and witness the outcomes of their choices in real time.

Therefore, this study aims to evaluate the effectiveness of combining GasMan software with CBL compared to traditional LBL in teaching inhalation anesthesia. The objective is to provide evidence supporting potential reforms in the curriculum for inhalation anesthesia courses.

## 2 Materials and methods

### 2.1 Study design and participants

Ethics approval for this study was obtained from the Education Committee of Guizhou Medical University (Approval No. JG2021026). All participants provided informed consent prior to participation, ensuring they were fully aware of the study’s objectives, procedures, and their rights to withdraw at any time without any consequences.

Participants were fourth-year students enrolled in the five-year anesthesiology program at Guizhou Medical University, from two consecutive academic cohorts: 2022 and 2023. Due to logistical constraints, students were divided by academic year into two groups: the 2022 cohort formed the traditional lecture-based learning (LBL) Group, while the 2023 cohort comprised the Integrating GASMAN software with case-based learning (IGC) Group. Although randomization was not employed, we used identical pre-course tests to control for baseline knowledge and ensure comparability between the groups.

### 2.2 Teaching method

Both groups shared identical learning objectives, encompassing a comprehensive understanding of inhalational anesthetics, including pharmacology, pharmacodynamics, pharmacokinetics, clinical application, and safety considerations. Below is a detailed description of the teaching methods for each group:

LBL group:

Participants: 110 students from the 2022 cohort.

Teaching method: Traditional lecture-based learning.

Course structure: The course consisted of 4 sessions, each lasting 45 min.

Content delivery: All sessions were delivered as theoretical lectures designed according to the curriculum requirements of the “Pharmacology of Anesthetics” textbook, as mandated by the National Health Commission’s “13th Five-Year Plan.”

Instructional tools: PowerPoint (PPT) presentations combined with teacher explanations.

IGC group:

Participants: 131 students from the 2023 cohort.

Teaching method: A hybrid approach integrating GASMAN software simulations with CBL.

Course structure: The course also consisted of 4 sessions, each lasting 45 min.

Content delivery:

Theoretical lectures: The theoretical content was streamlined into 2 sessions, similar to the LBL group, utilizing PPT presentations and teacher explanations.

Practical sessions: The remaining 2 sessions were designated for practical application. These sessions involved CBL where the instructor selected clinical cases that were representative and educationally valuable. The GASMAN software was used to simulate the absorption, distribution, and metabolism of inhalational anesthetics, reinforcing the theoretical concepts covered in the lectures.

In-Class Application: During the practical sessions, the GASMAN software served as a teaching aid. The instructor initially used PowerPoint presentations to introduce relevant clinical cases and explain the associated pharmacology, pharmacodynamics, and pharmacokinetics concepts. Then, students were divided into groups and used the GASMAN software to simulate different scenarios based on the clinical cases. Through the software’s dynamic simulation features, students could observe how inhalational anesthetics behave in the body under various conditions (e.g., different ages, weights, pathological states). This process aimed to provide students with a visual understanding of how inhalational anesthetics are absorbed and metabolized. After each simulation, student groups presented their observations, and the instructor guided the discussion with targeted questions to reinforce key concepts and deepen students’ comprehension of inhalational anesthetics.

Independent Exploration Outside of Class: To encourage further exploration outside of the classroom, students were assigned homework involving the independent use of the GASMAN software. They were tasked with simulating different patient profiles (e.g., those with heart failure, respiratory impairments) and adjusting drug dosages and ventilation parameters to observe how these variables affect anesthetic behavior. These out-of-class exercises were designed to foster independent learning and to enhance the students’ understanding of complex clinical scenarios.

### 2.3 Pre-course test

Prior to the commencement of the course, both groups of students were required to take a pre- course test on foundational knowledge of inhalational anesthesia. This test was administered without any prior preparation to accurately assess the students’ baseline understanding of the subject matter. The pre-class test scores served as the baseline data, providing insights into the students’ existing knowledge of inhalational anesthesia before any instructional intervention.

Test details:

Content coverage: The test covered essential inhalational anesthesia knowledge that is critical for clinical practice. This included the pharmacodynamics and pharmacokinetics of inhalational anesthetics, mechanisms of action, clinical application, and safety considerations.

Question source: The questions were randomly selected by a computer from the question bank accompanying the “Pharmacology of Anesthetics” textbook, which is part of the “13th Five-Year Plan” educational materials endorsed by the National Health Commission.

Question format: The test comprised 50 objective questions, each valued at 2 points.

Scoring: The total possible score was 100 points.

### 2.4 Teaching evaluation

#### 2.4.1 Teaching experts assessments

To assess the effectiveness of the teaching methods used in both the lecture-based learning (LBL) group and the IGC group, three highly experienced teaching experts in anesthesiology were selected. These experts, all holding senior academic titles, conducted evaluations based on standardized classroom teaching assessment criteria (detailed evaluation criteria can be found in Supplementary Material 1). The evaluation focused on the following five aspects:

Teaching design: Assessed the clarity of learning objectives, logical content flow, and appropriateness of teaching strategies, scored on a 0–20 scale.

Teaching implementation: Evaluated how well the teaching was delivered, including clarity of explanation and student engagement, scored on a 0–20 scale.

Classroom ambiance: Measured the level of student interaction, participation, and overall classroom dynamics conducive to learning, scored on a 0–20 scale.

Teaching effectiveness: Judged whether students met the learning objectives, with a focus on understanding and practical application, scored on a 0–20 scale.

Teacher quality: Assessed the teacher’s knowledge, communication skills, and ability to engage and motivate students, scored on a 0–20 scale.

Each of these five aspects was rated independently by the three experts, and the scores for each aspect were summed to provide a total score out of 100 points.

#### 2.4.2 Students evaluation

At the end of the course, students were asked to complete an anonymous paper-based questionnaire (see Supplementary Material 2) to evaluate the teaching methods. The questionnaire contained ten items, each rated on a 0 to 10 scale, with higher scores indicating a more favorable perception of the teaching method’s effectiveness. Anonymity was emphasized to ensure honest feedback and data validity.

Comprehending and mastering theoretical knowledge: Assessed how well the teaching method facilitated students’ comprehension and retention of theoretical concepts.

Improvement of practical skills: Evaluated the effectiveness of the teaching method in enhancing students’ practical skills relevant to clinical practice.

Resolving clinical challenges: Measured the ability of the teaching method to help students apply their knowledge to solve real-world clinical problems.

Nurturing clinical reasoning: Assessed how well the teaching method promoted the development of critical clinical thinking skills.

Increasing learning interest: Evaluated the extent to which the teaching method increased students’ interest and engagement in learning.

Enhancing learning efficiency: Measured how efficiently the teaching method helped students learn and retain information.

Consolidate memory: Assessed the effectiveness of the teaching method in helping students retain learned information over time.

Course logic: Evaluated the logical flow and coherence of the course as perceived by the students.

Improving analytical skills: Measured the ability of the teaching method to enhance students’ analytical skills.

Refining application proficiency: Assessed how well the teaching method helped students apply theoretical knowledge to practical situations.

Each item was rated on a scale from 0 to 10, with higher scores indicating a stronger perceived impact of the teaching method on that specific aspect. The total score for the evaluation was 100 points.

#### 2.4.3 Post-course tests

Upon completion of the course, students immediately participated in a post-course test to compare the effectiveness of the two teaching methods. The test items were drawn from the same test bank as the pre-course test but did not overlap with the pre-course questions. The total score for the post-course test was 100 points, with equal weight given to objective and subjective questions:

Objective Questions (50 points): These questions primarily assessed the students’ grasp of basic knowledge related to inhalation anesthesia. The objective section included 20 multiple-choice questions (each worth 2 points) and 10 true/false questions (each worth 1 point), designed to evaluate the students’ understanding of fundamental concepts.

Subjective Questions (50 points): This section aimed to evaluate students’ ability to apply theoretical knowledge in practical scenarios. It included 4 short-answer questions (each worth 5 points) and 3 case analysis and comprehensive application questions (each worth 10 points), requiring students to analyze, synthesize, and apply their knowledge to solve clinical problems.

### 2.5 Limitations

This study used cohort grouping, which may lead to baseline differences between the LBL and IGC groups. Although this design was dictated by logistical constraints, we controlled for initial academic performance and background characteristics to mitigate potential biases. Additionally, student evaluations may be subject to subjectivity; therefore, a combination of expert assessments and anonymous feedback was employed to enhance reliability. Future studies should consider a randomized design to improve the generalizability and robustness of the findings.

### 2.6 Data analysis

Statistical analyses were performed using SPSS software version 27.0. Categorical data (e.g., gender) were presented as frequencies and percentages, and group differences were evaluated using the chi-square test. Continuous variables were first assessed for normality using visual inspection of histograms. Variables with a normal distribution (e.g., age, pre-course test scores, student evaluation scores, post-course test scores) were summarized as mean ± standard deviation (SD), and comparisons between groups were conducted using an independent-samples *t*-test. Variables not conforming to a normal distribution (e.g., Expert Evaluation Scores) were summarized as medians and interquartile ranges (IQRs), and group differences were analyzed using the Mann–Whitney U test. For multiple comparisons, the false discovery rate (FDR) method was applied to adjust the *p*-values to control for type I error. A *p*-value of less than 0.05 was considered statistically significant for all analyses. As there were no missing data in this study, no data deletion or imputation was required.

## 3 Results

### 3.1 Pre-course tests

The demographic characteristics and pre-test scores of students in the IGC group and the LBL group showed no significant differences (*P* > 0.05). Specifically, the gender distribution was 58.8% female in the IGC group and 65.5% female in the LBL group (*P* = 0.288). The average age was 22.8 ± 0.9 years for the IGC group and 22.7 ± 1.0 years for the LBL group (*P* = 0.639). The pre-course test scores were 34.4 ± 10.1 for the IGC group and 33.2 ± 9.5 for the LBL group (*P* = 0.359) (Table 1).

**TABLE 1:** Baseline characteristics and pre-course test scores of students in the IGC and LBL groups.

	IGC group	LBL group	*P*-value
	**(*n* = 131)**	**(*n* = 110)**	
Sex, female, *n* (%)	77 (58.8)	72 (65.5)	0.288
Age, year, mean ± SD	22.8 ± 0.9	22.7 ± 1.0	0.639
Pre-course test, mean ± SD	34.4 ± 10.1	33.2 ± 9.5	0.359

### 3.2 Evaluation by teaching experts

The IGC group initially demonstrated higher scores than the LBL group in teaching design, classroom ambiance, teaching effectiveness, and total expert evaluation score, with statistically significant differences observed prior to applying the FDR adjustment (*P* < 0.05). However, after FDR correction, none of these differences reached statistical significance (adjusted *P* > 0.05). Consistently, there were no significant differences in teaching implementation or teacher quality between the two groups, both before and after correction (*P* > 0.05) (Table 2).

**TABLE 2:** Expert evaluation scores of teaching methods in the IGC and LBL groups.

	IGC group (*n* = 3)	LBL group (*n* = 3)	*U*-value	*P*-value	FDR adjust *P*
	**Median (IQR)**	**Median (IQR)**			
Teaching design	18.0 (17.5–18.5)	15.0 (14.5–15.0)	9.00	0.046	0.092
Teaching implementation	18.0 (17.5–18.0)	17.0 (16.5–17.5)	6.50	0.346	0.415
Classroom ambiance	18.0 (17.5–18.0)	15.0 (15.0–15.5)	9.00	0.043	0.258
Teaching effectiveness	18.0 (17.5–18.0)	15.0 (15.0–15.5)	9.00	0.043	0.129
Teacher quality	18.0 (18.0–18.5)	18.0 (17.5–18.5)	5.50	0.637	0.637
Total expert evaluation score	91.0 (88.0–91.0)	80.0 (78.5–80.5)	9.00	0.046	0.069

### 3.3 Student evaluation of teaching method

Students in the IGC group rated the teaching methods higher in several areas compared to the LBL group, with statistically significant differences in aspects such as comprehending and mastering theoretical knowledge, resolving clinical challenges, nurturing clinical reasoning, increasing learning interest, enhancing learning efficiency, consolidate memory, course logic, improve analytical skills, and refining application proficiency (adjusted *P* < 0.05). No significant differences were found in the improvement of practical skills and course logic (adjusted *P* > 0.05) (Table 3).

**TABLE 3:** Student evaluation scores for teaching methods in the IGC and LBL groups.

	IGC group (*n* = 131)	LBL group (*n* = 110)	Cohen’s d	*P*	FDR adjust *P*
	**Mean ± SD**	**Mean ± SD**			
Comprehending and mastering theoretical knowledge	7.4 ± 1.8	6.4 ± 2.3	2.04	< 0.001	< 0.001
Improvement of practical skills	6.5 ± 1.8	6.5 ± 1.8	1.77	0.818	0.818
Resolving clinical challenges	8.0 ± 1.4	6.4 ± 1.7	1.58	< 0.001	< 0.001
Nurturing clinical reasoning	8.0 ± 1.5	6.7 ± 1.7	1.57	< 0.001	< 0.001
Increasing learning interest	7.9 ± 1.4	6.7 ± 1.6	1.47	< 0.001	< 0.001
Enhancing learning efficiency	7.9 ± 1.5	6.8 ± 1.6	1.52	< 0.001	< 0.001
Consolidate memory	7.8 ± 1.4	6.5 ± 1.7	1.57	< 0.001	< 0.001
Course logic	7.9 ± 1.6	7.0 ± 1.5	1.52	< 0.001	< 0.001
Improve analytical skills	8.0 ± 1.3	6.5 ± 1.6	1.48	< 0.001	< 0.001
Refining application proficiency	8.1 ± 1.5	6.0 ± 1.7	1.62	< 0.001	< 0.001
Total student evaluation score	77.5 ± 4.6	65.5 ± 5.1	2.49	< 0.001	< 0.001

### 3.4 Post-course test

In the post-course test, the IGC group had significantly higher total post-course test scores (74.9 ± 8.9 vs. 67.3 ± 8.9, adjusted *P* < 0.001) and subjective questions scores (38.3 ± 5.9 vs. 32.4 ± 5.4, adjusted *P* < 0.001) compared to the LBL group. No significant differences were observed in objective questions scores between the two groups (36.6 ± 7.1 vs. 35.0 ± 7.5, adjusted *P* = 0.089), (Table 4).

**TABLE 4:** Post-course test scores of students in the IGC and LBL groups.

	IGC group (*n* = 131)	LBL group (*n* = 110)	Cohen’s d	*P*-value	FDR adjust *P*
	**Mean ± SD**	**Mean ± SD**			
Objective questions	36.6 ± 7.1	35.0 ± 7.5	7.28	0.089	0.089
Subjective questions	38.3 ± 5.9	32.4 ± 5.4	5.67	< 0.001	< 0.001
Total post-course test score	74.9 ± 8.9	67.3 ± 8.9	8.93	< 0.001	< 0.001

## 4 Discussion

Our study investigates the effectiveness of integrating GASMAN software with case-based learning (CBL) in teaching inhalation anesthesia to undergraduate anesthesiology students. The results demonstrate notable improvements in teaching outcomes, particularly in areas involving clinical reasoning and subjective assessment, as evidenced by superior student evaluations and higher scores on subjective questions in post-course assessments, compared to the traditional lecture-based learning (LBL) approach. However, after applying the false discovery rate (FDR) correction, no statistically significant differences were found in expert evaluation scores between the IGC and LBL groups. This finding underscores the complexities of interpreting our results and suggests that while GASMAN-enhanced CBL may improve certain aspects of learning, it does not uniformly impact all areas of teaching.

Inhalation anesthesia education presents unique challenges due to the abstract and complex nature of pharmacokinetic and pharmacodynamic concepts (2, 18). Our study revealed that while the LBL group achieved an overall post-course test score rate below 70%, indicating the inherent difficulty of the material, objective test scores between the IGC and LBL groups showed no significant difference (adjusted *P* = 0.089). This lack of significant differences in objective assessments suggests that while GASMAN-enhanced CBL was effective in improving subjective understanding, it did not translate to better factual recall, which is often the focus of objective tests. This may indicate that different teaching methods impact distinct types of learning outcomes; CBL combined with GASMAN appears to enhance higher-order thinking skills and application-based understanding, rather than simple factual recall.

CBL is well-regarded in clinical education for its use of real-world scenarios to promote active learning and problem-solving (7). In our study, the IGC group demonstrated higher scores in subjective areas such as fostering clinical reasoning, increasing engagement, and enhancing analytical skills. The significant improvement in subjective test scores underscores the potential of CBL to deepen conceptual understanding and encourage critical thinking, skills essential for clinical practice. However, CBL alone may not fully address the inherent abstraction in inhalational anesthetics. The addition of GASMAN software, which simulates the pharmacokinetics of inhalational agents and allows users to manipulate parameters like body weight, cardiac output, and anesthetic concentrations, provided a visual and interactive platform to reinforce theoretical knowledge (10, 11). This dual approach seems particularly effective for fostering deep learning, as reflected in the IGC group’s higher evaluations of memory consolidation and learning efficiency compared to the LBL group. These findings suggest that the interactive features of GASMAN, such as real-time adjustments and visual feedback, play a crucial role in helping students grasp complex pharmacokinetic dynamics (11).

Despite the promising results, it is important to note that expert evaluations did not show statistically significant differences between the two groups after applying the false discovery rate (FDR) correction. Several factors may explain this lack of statistical significance. One possibility is the small number of expert evaluators (*n* = 3 per group), which may have limited the statistical power of these comparisons, resulting in non-significant findings after adjusting for multiple comparisons. A larger panel of evaluators with more diverse backgrounds could reduce scoring variability and lead to more robust findings. Additionally, this outcome may reflect the subjective nature of expert evaluations, which are influenced by personal experiences and expectations.

Furthermore, the lack of significant differences in objective test scores indicates that GASMAN-enhanced CBL may not universally enhance all aspects of knowledge acquisition. While GASMAN software appears effective in promoting conceptual understanding and clinical reasoning, it may not be as beneficial for memorizing factual content. This suggests that different teaching methods may be better suited for distinct learning objectives—whether the focus is on higher-order cognitive skills or basic factual knowledge. Future research could explore whether a more targeted integration of GASMAN, perhaps by incorporating additional drills or review sessions to reinforce factual content, might help bridge this gap.

Several practical considerations must also be taken into account when implementing GASMAN-enhanced CBL. Instructors need to be proficient not only in using the software but also in understanding the pharmacological principles it illustrates. Additionally, clinical cases used in the simulation should be carefully curated to align with educational objectives and replicate real-world scenarios. A well-structured teaching schedule is also essential, ensuring an appropriate balance between theoretical learning and practical application, so students can engage meaningfully with the software and reflect on their learning.

Lastly, our study has several limitations. The sample was restricted to undergraduate anesthesiology students from a single institution, and the assessment focused on short-term learning outcomes. Consequently, the long-term impact of GASMAN-enhanced CBL on clinical performance remains unknown. Future studies should aim to include a more diverse student population and extend the follow-up period to assess the durability of learning outcomes over time.

In conclusion, while our findings demonstrate that integrating GASMAN software with CBL can enhance the teaching of inhalational anesthesia, particularly in developing clinical reasoning and deep understanding, the intervention does not appear to uniformly improve all types of learning outcomes. A balanced approach that considers the strengths and limitations of each teaching method is essential for optimizing anesthesia education. This study contributes to the ongoing effort to innovate medical education, highlighting the need for adaptive, evidence-based teaching strategies in a field that is constantly evolving.

## Data Availability

The raw data supporting the conclusions of this article will be made available by the authors, without undue reservation.
