# Supplementary material for: Evaluation of the effectiveness of GASMAN anesthesia simulation software combined with case-based learning versus traditional lecture-based learning in inhalation anesthesia education
Source: Front Med (Lausanne). 2025 Jan 6;11:1472404. doi: 10.3389/fmed.2024.1472404 (PMC11743626; doi:10.3389/fmed.2024.1472404)
Supplement: Supplementary file 1 [file Data_Sheet_1.pdf]

Supplementary Material S1:

Evaluation Form for Classroom Teaching by Faculty of Anesthesiology (English Version)

Course Title:____________ Date:_________ Location:__________ Instructor:__________

Evaluation Criteria	Points Available	Score
**Teaching Design**		
1. Teaching Philosophy: The teaching philosophy reflects a "student-centered" approach, incorporates moral education, and aligns with the discipline's characteristics and course requirements.	4	
2. Teaching Objectives: The teaching objectives are clear, specific, easy to understand, and implementable. Behavioral verbs are used correctly, and the descriptions are standardized.	4	
3. Content Analysis:		
a. The teaching content has depth and breadth, reflects high-level, innovative, and challenging aspects.	2	
b. Reflects the cutting edge of the discipline, integrates professional ideas, and uses high-quality teaching resources.	2	
c. The teaching content meets industry and societal needs, addresses key and difficult points appropriately, and considers students' prior knowledge and experience.	2	
d. Connects teaching content with new developments in discipline research, practical experience, and societal changes.	2	
4. Teaching Plan: The overall design of the teaching plan is innovative and meets the requirements of the teaching objectives; the teaching process design has distinctive features.	4	
**Teaching Implementation**		
1. Teaching is organized, and the process is arranged reasonably.	4	
2. Understands students' learning characteristics in the modern era and fully utilizes modern information technology for course teaching activities and learning evaluation.	4	
3. Teaching activities are diverse and reflect the knowledge, skills, and emotional value goals at various levels.	3	
4. Creatively uses textbooks, content is substantial and concise, suitable for students' level; structure is reasonable, transitions are natural, and it is easy to follow; links theory with practice, inspires students to think and solve problems.	3	

5. Uses innovative teaching strategies, methods, and technologies to solve various problems and difficulties in the classroom based on the course characteristics; emphasizes key points and accurately handles difficult points.	3	
6. Reasonably selects and applies information technology, creates a teaching environment, focuses on interactions between teacher-student and student-student, and emphasizes autonomous, cooperative, and inquiry-based learning.	3	
**Classroom Ambiance**		
1. Innovates teaching methods and strategies, emphasizes teaching interaction, inspires students to think and solve problems.	4	
2. Students actively participate in learning, construct and organize knowledge independently.	3	
3. The classroom is flexible and open, focusing on cultivating students' creative thinking.	4	
4. The classroom involves broad participation, covering different student levels.	3	
5. The classroom atmosphere is harmonious, with positive teacher-student emotions and a democratic teaching approach.	3	
6. There is equal and multi-directional exchange of ideas between teachers and students, and among students.	3	
**Teaching Effectiveness**		
1. Classroom lectures are engaging, the classroom atmosphere is harmonious, and students are actively involved.	4	
2. Students' knowledge, abilities, and thinking are developed, and the teaching objectives are achieved.	4	
3. Forms a teaching model suitable for the discipline's characteristics and students' traits, with considerable reference and promotion value.	4	
4. Based on real classroom problems, reflects the "student development-centered" philosophy, and proposes solutions to problems.	4	
5. Uses multiple evaluation methods to reasonably assess students' knowledge, abilities, and thinking development.	4	
**Teacher Quality**		
1. Dignified demeanor, energetic, and clear language expression.	4	
2. Rich professional knowledge, understanding of the forefront of the professional field, thorough lesson preparation.	4	
3. Strong ability to organize and control the classroom.	4	
4. Effective use of visual teaching aids and modern educational technology.	4	

5. Good at creating scientific and interesting teaching situations to guide students in problem-solving.	4	
**Comments:**<br><br><br>Signature of the Evaluating Teacher:		Total Score:
