# Supplementary material for: Evaluation of the effectiveness of GASMAN anesthesia simulation software combined with case-based learning versus traditional lecture-based learning in inhalation anesthesia education
Source: Front Med (Lausanne). 2025 Jan 6;11:1472404. doi: 10.3389/fmed.2024.1472404 (PMC11743626; doi:10.3389/fmed.2024.1472404)
Supplement: Supplementary file 2 [file Data_Sheet_2.pdf]

Supplementary Material S2:

Faculty of Anesthesiology Teaching Feedback Questionnaire (English Version)

Course Title: **Instructor Name:**

Dear Students,

Thank you for participating in this course. To further improve the quality of our teaching, we would like to gather your feedback on this course. Please rate each aspect on a scale from 0 to 10, with higher scores indicating greater satisfaction and benefit from the course.

1. **Comprehending And Mastering Theoretical Knowledge**
   Did this course help you better understand and master the relevant theoretical knowledge?

   Rating: 0 | 1 | 2 | 3 | 4 | 5 | 6 | 7 | 8 | 9 | 10

2. **Improvement of Practical Skills**
   How much did this course help you improve your clinical practical skills?

   Rating: 0 | 1 | 2 | 3 | 4 | 5 | 6 | 7 | 8 | 9 | 10

3. **Ability to Solve Clinical Problems**
   Did this course help you develop the ability to solve actual clinical problems?

   Rating: 0 | 1 | 2 | 3 | 4 | 5 | 6 | 7 | 8 | 9 | 10

4. **Nurturing Clinical Reasoning**
   Did this course promote the development of your clinical reasoning and critical thinking skills?

   Rating: 0 | 1 | 2 | 3 | 4 | 5 | 6 | 7 | 8 | 9 | 10

5. **Increased Interest in Learning**
   Did this course stimulate your interest and enthusiasm for learning?

   Rating: 0 | 1 | 2 | 3 | 4 | 5 | 6 | 7 | 8 | 9 | 10

6. **Improved Learning Efficiency**
   Did you find that this course improved your learning efficiency and memory retention?

   Rating: 0 | 1 | 2 | 3 | 4 | 5 | 6 | 7 | 8 | 9 | 10

7. **Consolidate Memory**
   Did this course help you better consolidate and remember the knowledge you have learned?

Rating: 0 | 1 | 2 | 3 | 4 | 5 | 6 | 7 | 8 | 9 | 10

8. **Course Logic and Coherence**
   How would you rate the logical flow and coherence of this course?

   Rating: 0 | 1 | 2 | 3 | 4 | 5 | 6 | 7 | 8 | 9 | 10

9. **Improved Analytical Skills**
   Did this course enhance your analytical and problem-solving skills?

   Rating: 0 | 1 | 2 | 3 | 4 | 5 | 6 | 7 | 8 | 9 | 10

10. **Refining Application Proficiency**
    How much did this course help you apply theoretical knowledge to practical clinical situations?

    Rating: 0 | 1 | 2 | 3 | 4 | 5 | 6 | 7 | 8 | 9 | 10

Thank you for your valuable feedback!
